# IL8 and Cathepsin B as Melanoma Serum Biomarkers

**DOI:** 10.3390/ijms12031505

**Published:** 2011-02-25

**Authors:** Hongtao Zhang, Ting Fu, Suzanne McGettigan, Suresh Kumar, Shujing Liu, David Speicher, Lynn Schuchter, Xiaowei Xu

**Affiliations:** 1 Department of Pathology and Laboratory Medicine, University of Pennsylvania, Philadelphia, PA 19104, USA; E-Mails: tingfu@mail.med.upenn.edu (T.F.); sureshmk@mail.med.upenn.edu (S.K.); shujing@mail.med.upenn.edu (S.L.); xug@mail.med.upenn.edu (X.X.); 2 Division of Hematology-Oncology, HUP, 16 Penn Tower, Philadelphia, PA 19104, USA; E-Mails: Suzanne.McGettigan@uphs.upenn.edu (S.M.); Lynn.Schuchter@uphs.upenn.edu (L.S.); 3 The Wistar Institute, 3601 Spruce St., Philadelphia, PA 19104, USA; E-Mail: speicher@wistar.org

**Keywords:** IL-8, cathepsin B, melanoma, serum interference

## Abstract

Melanoma accounts for only a small portion of skin cancer but it is associated with high mortality. Melanoma serum biomarkers that may aid early diagnosis or guide therapy are needed clinically. However, studies of serum biomarkers have often been hampered by the serum interference that causes false readouts in immunological tests. Here we show that, after using a special buffer to eliminate the serum interference, IL-8 and cathepsin B levels were significantly elevated in melanoma patients (*p* < 0.05). More importantly, the combination of IL-8 and cathepsin B were also studied as a prognosis marker for melanoma mortality. Our study provides a novel approach to examine serum biomarkers.

## 1. Introduction

Melanoma is a malignancy that arises from melanocytes in the skin and eye. According to the American Cancer Society, melanoma of skin is the 5th most common cancer in the USA and accounted for 4–5% of newly diagnosed cancer cases in 2009. The incidence of this cancer has been increasing for more than 30 years, and the number of melanoma cases worldwide is increasing faster than any other types of cancer [1]. Although melanoma only represents a very small portion of all skin cancers, it is responsible for the majority of skin cancer deaths. Early detection of melanoma has led to an increase of the 5-year relative survival rates from ∼80% in 1975 to >90% in 1996 [2].

Most of the melanomas retain melanocytic differentiation markers such as S-100, Melan-A, tyrosinase, tyrosinase related proteins (TRPs), Microphthalmia, HMB-45, and so forth [3]. These markers (except for S-100) are very specific and are used clinically to confirm melanocytic differentiation by immunohistochemical staining. The serum level of 5-s-cysteinyldopa (5-SCD), a pheomelanin precursor, has been reported to correlate with the progression of melanoma [4], although it is also elevated in some other patients with skin hyperpigmentation, such as patients with renal disease and subject to hemodialysis [5]. S100A has been studied as a biomarker to predict relapse and death. Immunohistochemical analysis indicates that certain subtypes, such as S100BB, are associated with increased risk of relapse [6]. Lower serum S100A1B and S100BB have also been associated with better survival. However, currently only serum lactate dehydrogenase (LDH) is clinically used as a biomarker for late stage melanoma as recommended by the American Joint Committee on Cancer tumor, node, metastasis staging system [7]. There is a clear unmet medical need to develop other melanoma serum biomarkers for disease progression. A large number of serum proteins have been screened in proteomic studies and novel approaches have been applied to study these proteins [8–10].

We previously discovered a supersensitive immune detection assay to analyze low abundant antigens in sera [11]. We have since studied several molecules, including cytokines and melanocyte specific antigens in the sera of patients with melanoma. To our surprise, detection of many of these proteins was not hampered by the sensitivity of the assay but by the serum interference that causes false positive signals. The culprit for the serum interference was the presence of high levels of heterophilic human anti-animal immunoglobulin antibodies (HAIA), such as human anti-mouse antibodies (HAMA), in certain populations of people [12]. HAIA is produced by human immune reaction following an exposure to mice or other animal agents, including murine monoclonal antibodies associated with some therapeutic or diagnostic procedures [13]. Some human auto-antibodies, the most common of which is rheumatoid factor (RF), also bind to animal antibodies by cross reactivity. The prevalence of HAIA is varied in different reports (reviewed in [12]). In one study, human anti-bovine antibodies were detected in 99% of the donor serum samples [14].

Since HAIA represents a big interference for ELISA assays on serum samples, many approaches have been proposed to reduce the effect of these agents. Maher *et al.* reported that HAMA was no longer an issue if immunoglobulin- free fraction of serum was used for immunoassays [13]. The strategy, however, was not practically sound considering the number of samples and the cost of the procedure. Mouse IgG derived blocking reagents, such as Immunoglobulin Inhibiting Reagent (IIR; Bioreclamation), Heterophilic Blocking Reagent (HBR; Scantibodies), Heteroblock (mixture of active and passive blocking reagents; Omega Biologicals), and MAB 33 (monoclonal IgG1) and Poly MAB 33 (polymeric monoclonal IgG1/Fab; Boehringer Mannheim), have also been developed for HAMA positive samples, but the results varied [15]. A common practice in the immunoassay for a suspected sample is to use alternative detection antibodies of different species. Yu *et al.* reported false-positive detection of extremely high levels of the C-reactive protein (CRP) when goat-anti CRP antibody was used to test the serum from a subject who had no corresponding symptoms [16]. In this case, the culprit was identified as the monoclonal IgM-λ paraprotein that had bind to the goat antibody. The use of chicken antibodies appears to avoid the HAMA interference in some assays [17,18].

In this report, we have examined the serum concentrations of several potential melanoma biomarkers using an approach that eliminates the serum interference. Previously, elevated IL-8 level was associated with metastatic melanoma [19], and decrease of serum IL-8 levels were correlated with response to chemotherapy or immunochemotherapy [20]. In a proteomic analysis, elevated cathepsin B level was detected from the sera of mice carrying human melanoma xenografts [21]. Here, our studies indicate that the IL-8 level is significantly elevated in melanoma but not in breast cancer patients, while the Cathepsin B level is higher in both melanoma and breast cancer as compared with healthy subjects. Like LDH, the combination of IL-8 and Cathepsin B serum levels can be used to predict the medium term mortality (3–5 years) of melanoma patients. The potential implication of our observation is discussed.

## 2. Results and Discussion

### 2.1. Optimization of ELISA Assay

**Figure 1.**
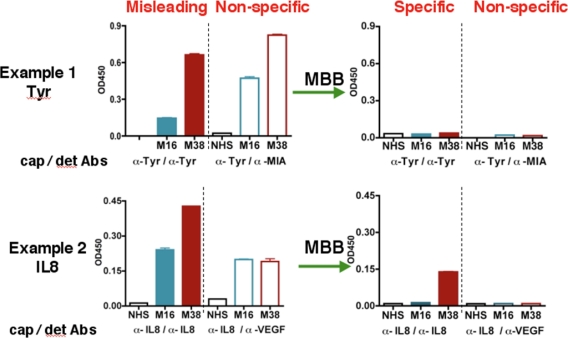
MBB reduces non-specific bindings in the ELISA assay. Serum samples NHS, M16, and M38 were diluted in either 1% BSA (left) or MBB buffer (right) for ELISA assay. For Tyrosinase (Tyr), antibody T9230 (US Biologicals) and biotinylated T311 were used as the capture antibody and the detection antibody, respectively. For IL-8, the IL-8 Cytoset from Biosource was used. To show non-specific bindings, Tyr or IL-8 capture antibodies were used but biotinylated anti-MIA or anti-VEGF antibodies were used respectively as the detection antibodies.

During the course of studying serum biomarkers, we encountered problems with certain samples that suspiciously showed elevated levels for almost all the targets we tested. Two examples were shown in Figure 1 (left panel). Following the suggested conditions in commercially developed ELISA kit, we detected “high concentrations” of both IL-8 and Tyrosinase (Tyr) in the serum samples M38 and M16. M38 was obtained from a melanoma patient but unexpectedly M16 was from a healthy volunteer. The sandwich ELISA used an unmodified capture antibody and a biotinylated detection antibody for each antigen. The tertiary antibody-antigen complex was detected by the biotin-binding streptavidin-HRP conjugate. To test whether the signal was antigen-specific, we designed a control assay using non-matching antibody sets, e.g., a MIA detection antibody paired with the Tyr capture Ab, or a VEGF detection Ab paired with the IL-8 capture Ab (Figure 1, the “non-specific” panel). The results showed strong signals in both samples, indicating that previous ELISA readings of these samples under conventional conditions were erroneous and misleading.

The heterophilic human anti-animal immunoglobulin antibodies (HAIA) may account for the misleading results in the ELISA assay [22]. If the serum samples containing HAIA recognizes the Fc region of the capture and detection antibody, it will crosslink these two antibodies, leading to false-positive signals in the absence of the true antigen. On the other hand, if the existing HAMA binds to the Fv regions of the capture or detection antibody, it will prevent the true antigen from binding to the detection or capture antibody, resulting in a false-negative signal.

After initial unsuccessful experience with several commercial serum interference inhibitors, we have identified a buffer named MBB (Martell Biosystems, Inc.) that eliminates the false readings. HAIAs are generally less specific and have lower affinity towards the target. The MBB buffer is designed to prevent the weak interaction between HAIA and the target but not the strong interaction between capture/detection antibodies and specific antigens.

In the “standard” ELISA, serum samples were diluted 1:10 with 1% BSA. When MBB buffer was used to dilute the serum samples, the non-specific binding, as revealed by the non-matching pairs of antibodies, disappeared (Figure 1, right panels). The MBB buffer was thus applied to the ELISA to measure the specific binding to targets. Sample M16 from healthy volunteer was no longer positive for Tyr or IL8 in the presence of MBB. In contrast, the melanoma sample M38 was still positive for IL-8. Clearly, the readouts under the conventional conditions were influenced by the HAIA in the serum and did not reflect the true concentrations of these antigens. We believe the modified ELISA assay using MBB provides the true value for serum concentrations of target proteins.

### 2.2. Analysis of Serum IL-8

**Figure 2.**
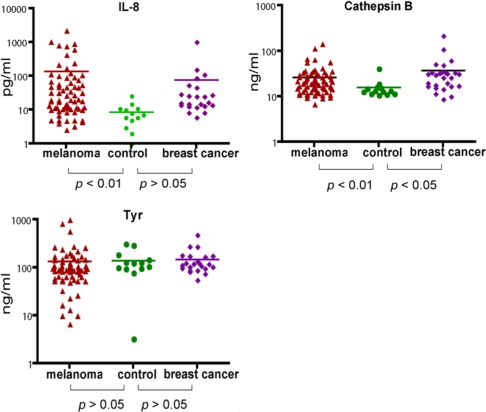
Comparison of serum cathepsin B, IL-8 and Tyrosinase in melanoma, breast cancer and healthy controls.

Using the MBB buffer to eliminate the serum interference, we have analyzed several serum markers that may associate with melanoma. First we studied IL-8, a cytokine secreted by melanoma cells. Serum levels of IL-8 were significantly higher in melanoma patients (*p* < 0.01) (Figure 2). The elevated IL-8 levels were independent of age (Table 1). A trend of higher IL-8 levels in later stages of melanoma was observed (Table 1) but the difference was not statistically different. In a study of a group of breast cancer patients, the average IL-8 levels in breast cancer was higher than that of control, but the difference was not statistically different.

### 2.3. Analysis of Serum Cathepsin B

Cathepsin B was found to be elevated in the sera of mice carrying human melanoma xenografts [21]. Cathepsin B is a lysosomal enzyme that is involved in cancer invasion and metastasis [23]. We thus examined if serum cathepsin B from melanoma patients can be used as a biomarker to predict the progress of the disease. Indeed, significantly elevated levels of Cathepsin B (as determined by unpaired *t* test) were observed in melanoma patients. However, Cathepsin B levels were also elevated in breast cancer patients, indicating that cathepsin B is not unique to melanoma but may be associated with general tumor progression. Cathepsin B levels were also independent of age and stages of melanoma (Table 2).

### 2.4. Analysis of Serum Tyrosinase

Tyrosinase, a type I membrane protein and copper-containing enzyme, is involved in the production of melanin. Melanin biosynthesis requires the enzymatic activity of tyrosinase, which catalyzes the critical and rate limiting step of tyrosine hydroxylation [24]. Defects affecting tyrosinase activity result in various forms of albinism [25]. We examined the serum levels of tyrosinase. Despite detection of extremely high levels of tyrosinase in the serum of several melanoma patients, there was no difference in the average levels between melanoma and controls. Breast cancer samples also appeared to have baseline levels of tyrosinase. Our data indicate that serum tyrosinase is not an effective biomarker for melanoma or breast cancer.

### 2.5. Analysis of Additional Cytokines

Although in general melanoma patients have higher serum IL-8 and cathepsin B levels, neither of these two proteins is expected to serve as a single melanoma biomarker. For example, using 2 × SD above the average of the control group as the cut-off value, we determined that the sensitivity of serum IL-8 for melanoma is just about 46%. The sensitivity of cathepsin is even lower. In addition, elevated levels of these proteins have been observed in other cancer patients, leading to lower specificity for both proteins as melanoma biomarkers. However, it is suggested that IL-8 and cathepsin B could be used together and incorporated into a melanoma biomarker panel. To study if additional serum proteins can also be used to characterize melanoma, we also performed multiplex ELISA assays in MBB buffer using the Milliplex cytokine/chemokine kit for 39 human target proteins (Millipore) for sera from 11 melanoma patients. We found that nine cytokines/chemokines showed statistically higher levels in melanoma patients compared to controls (Table 3).

### 2.6. Serum Biomarkers for Melanoma Specific Death

Clinical follow up data were available on these patients for up to 3 years post serum collection. We noticed a higher mortality rate for patients who had extremely high levels of these circulating proteins: 100% (2 out of 2) for cathepsin B, 66.7% (6 out of 9) for IL-8, and 75% (3 out of 4) for tyrosinase. To further study whether these serum markers may predict prognosis, melanoma patients were divided into the High mortality group or the Low mortality groups depending on their status during the follow-up for 3 years. For all patients who suffered from melanoma-related death within 3 years after enrollment of the study, their serum samples collected at the beginning of the study were assigned to the High Mortality group. Samples from other subjects who survived for more than 3 years after enrollment of the study were allocated into the Low mortality group. Both groups consist of patients with diversified disease stages (II–IV). Some patients did not have measurable disease at the time of serum collection. The initial serum levels of investigated target proteins were compared between these two groups. The serum levels were considered elevated if they were above the cut-off value, which was determined using the average value of controls plus 2 standard deviation (average ± 2 × SD).

Unexpectedly, none of these biomarkers (IL-8, Cathepsin B, or tyrosinase) can be used as an independent biomarker to differentiate the High and Low mortality groups (*p* > 0.05, data not shown). A modest increased of mortality was observed in patients with high IL-8 levels. The mortality was 62.9% in patients with elevated IL-8 levels, and 48.7% in patients with normal IL-8 levels. This resulted in a risk ratio of 1.29, with 95% confidence interval (CI) ranging from 0.86 to 1.95 (*p* > 0.05). There was also a higher risk of mortality for patients with elevated cathepsin B levels (risk ratio = 1.38) but the difference was not statistically significant (Fisher’s exact test, *p* > 0.05).

**Figure 3.**
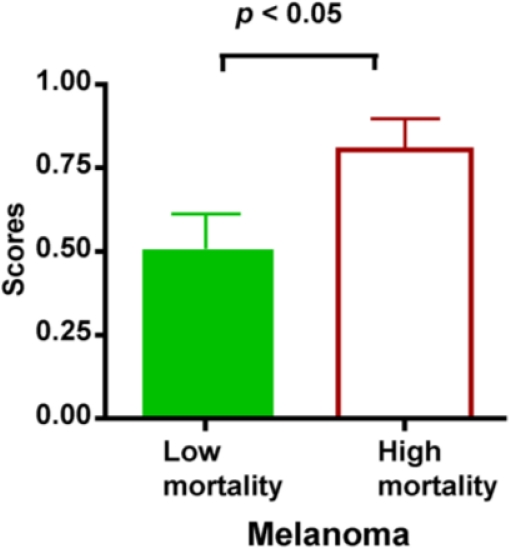
Biomarker scores can be used for the prognosis of melanoma. Serum samples from melanoma patients were tested for elevated levels of IL-8 and Cathepsin B. High mortality group: patients died within 3 years after enrollment of the study; Low mortality group: patients survived for more than 3 years after enrollment of the study. Patients were scored based on the initial serum levels of IL-8 and Cathepsin B. Patients receive 0 score for normal levels, 1 score for elevated levels of only one biomarker and 2 scores if both were elevated. Patients in the High mortality group showed significantly higher scores as judged by unpaired t test. Data were presented as mean ± SEM.

To investigate if the combination of IL-8 and Cathepsin B can be used for predicting mortality, we established a biomarker scoring system and assigned 1 score for a patient if he had elevated level of any of these two biomarkers. A patient received at most 2 scores when he had elevated levels for both IL-8 and Cathepsin B. Using such a scoring system, we were able to show that patients in the High mortality group had significantly higher scores (*p* < 0.05) (Figure 3).

**Figure 4.**
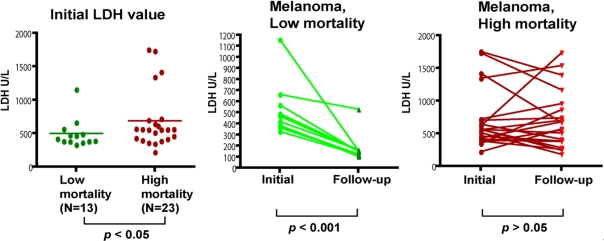
LDH as a prognosis marker for melanoma. Records of LDH levels of patients at the time of serum sample donation as well as 18-months later (or the last available LDH record if the patient died before 18-months follow-up) were examined. Patients in the High mortality group had significantly higher levels of LDH than those in the Low mortality group. The Low mortality group also showed significant reductions in LDH levels in the follow-up (paired *t* test, *p* < 0.001), while the High mortality group experienced no detectable changes in the LDH levels.

For melanoma, Lactate dehydrogenase (LDH) is not a specific marker but has been used as a prognostic marker for metastasis [7]. We also retrospectively examined all the available LDH records for patients we have for this study. First we looked at the LDH levels at the time when the patients donated their serum samples for the study. Patients in the High mortality group demonstrated significantly higher levels of LDH than those in the Low mortality group (674.1 ± 80.3 U/L *vs.* 471.2 ± 38.1 U/L, *p* = 0.04). This confirms the significance of LDH as a prognosis marker for melanoma. Furthermore, we also examined the follow-up LDH value at 18 months after the initial visit, or the last available value if patients were deceased before 18 months. The Low mortality group showed significant reductions in LDH levels in the follow-up (paired *t* test, *p* < 0.0001), while the High mortality group experienced no detectable changes in the LDH levels (*p* = 0.76) (Figure 4). Our data indicated that the change of LDH could be used as a much better biomarker than the baseline level for the prognosis of melanoma related death.

Unfortunately, we did not collect follow-up serum samples from these patients for the analysis of IL-8, Cathepsin B or Tyrosinase. It is not clear if melanoma patients experience changes in the serum of these proteins and if that change can be used as an accurate biomarker for prognosis.

### 2.7. Discussion

Despite the reports of a variety of serum proteins as potential biomarkers for the diagnosis and prognosis of melanoma, few can be confirmed in large scale studies. According to the joint committee of tumor staging, LDH is the only marker that can clinically help characterize melanoma. Although many factors may account for this, one issue that cannot be ignored is the false positives that inflate the variation of test results among already complex samples. As we have experienced in the study of biomarkers using immunological approaches, the use of serum proteins as biomarkers have been hampered by the unpredictable serum interference due to the presence of human anti-animal immunoglobulin antibodies (HAIA).

We have shown that by using a special buffer to eliminate serum interferences, two serum proteins, IL-8 and Cathepsin B, were significantly elevated in melanoma patients. Although the analysis of breast cancer samples indicated that elevated IL-8, not Cathepsin B, was associated with melanoma, both proteins are not unique to melanoma. To our surprise, Tyrosinase, a melanocyte specific marker that is involved in pigment synthesis [24], appears to maintain a normal level in the majority of melanoma patients as compared to healthy or breast cancer patients. We also studied MIA, another melanocyte specific marker, and unexpected found that only a small number of melanoma patients have elevated serum levels (data not shown). Furthermore, using multiplex assay, we have identified additional cytokines/chemokines that have higher serum levels in melanoma.

There are clear evidences that IL-8 is associated with melanoma progression. IL-8 is produced by melanoma cell lines and functions as an autocrine growth factor [26]. Elevated IL-8 levels have been observed in patients with metastatic melanoma [19]. Higher blood levels of IL-8 and IL-6 may be associated with the Brenner sign, which is an erythematous eruption in the vicinity of or distant from the lesion in melanoma patients [27]. In a group of stage IV melanoma patients, decreased serum IL-8 levels are found in patients with response to chemotherapy or immunochemotherapy [20]. By blocking IL-8, the anti-IL8 antibody (ABX-IL8) effectively reduced the growth of melanoma xenografts as well as metastasis of melanoma cells [28]. Due to the importance of its involvement in the development and metastasis of melanoma, IL-8 has been studied frequently together with other pro-inflammatory cytokines to identify potential clinical biomarkers for diagnosis and prognosis [29].

Cathepsin B is a cysteine protease that has been found upregulated in aggressive human pancreatic endocrine neoplasms [23]. Secreted active cathepsin B was detected from both preneoplastic and malignant cells [30]. Cathepsin B may play a role in the promoting of tumor cell invasiveness, as cathepsin B deficiency resulted in reduced tumor vascularity and invasion in mice [23]. Knocking down of cathepsin B by RNAi also led to comprised glioma cell invasion and angiogenesis both in vitro and in vivo [31].

In our study, serum cathepsin B levels were elevated in both melanoma and breast cancer patients. Based on these data, it is suggested that cathepsin B might be relevant to the development of a broad range of tumor, possibly contributing to the tumor cell migration and metastasis due to its protease function. Interestingly, IL-8 and cathepsin B, the two proteins we have been focusing on in our study, appeared to have somehow linked functions. In a study of endothelial cell migration mediated by IL-8, cathepsin B was found to be critical for IL-8 induced transactivation of EGFR [32].

More importantly, our study suggests that serum IL-8 and cathepsin B levels can potentially be used as prognostic markers for melanoma. In fact, the combination of IL-8 and cathepsin B into a scoring system has been shown to differentiate deceased patients from those who survived the 3 year follow-up. However, we understand that additional studies are required to further confirm the use of IL-8/Cathepsin B as prognosis markers. In this initial study, patients are not controlled for treatments. We are planning to examine the changes of IL-8 and Cathepsin B levels over the course of controlled treatments. It will be clinically useful if IL-8/Cathepsin B can be used to predict the treatment response in patients so unresponsive patients may consider other treatment options as early as possible. We expect that further study could verify if any additional serum proteins, such as those identified in our multiplex study, can be incorporated into the scoring system to establish the melanoma biomarker panel for both diagnosis and prognosis.

## 3. Experimental Section

### 3.1. Patients

Melanoma patients (stages II–IV, *n* = 67) were consecutive patients diagnosed with melanoma through the oncology clinic at the University of Pennsylvania. Patients did not all have measurable disease at the time they donated serum samples for the study. In addition, we also studied sera from patients with breast cancers (*n* = 24) to test whether the changes were specific for melanoma. Control participants were healthy volunteers. All participants were recruited according to a protocol approved by the institutional review board (IRB) and informed that this study is purely investigational in nature and that results of individual tests will not be made available to them.

### 3.2. ELISA Assay with MBB Buffer

ELISA kit for IL-8 (Biosource, Catalog # CHC1303) and Cathepsin B (R&D Systems, Catalog # DCATB0) were purchased. For ELISA, the capture antibodies (anti-IL-8, Biosource clone 893A6G8), anti-Tyr (Santa Cruz, # 9230), and anti- cathepsin B (R&D, Clone155714) were coated in carbonate-bicarbonate coating buffer (pH 9.6, 1 μg/mL, 50 μL) to a 96-well plate for overnight incubation at 4 °C. After wash with PBST, the plate was blocked with 1% casein or 5% BSA for 1 h at 22 °C. Protein standard and serum samples were diluted in the blocking buffer containing BSA or MBB (Martell Biosystem) and incubated in the blocked plate (50 μL per well) for a 1-h incubation at 22 °C. Diluted biotinylated detection antibody (50 μL, 180 ng/mL, with MBB) was added to each well and incubated for 1 h. Detection antibodies we used were: anti-IL8: clone 790A28G2 (Biosource), anti-Tyr: Clone T311 (Neomarkers). Streptavidin-conjugated HRP was used as the secondary antibody to detect the antigen-antibody complex. The Streptavidin-HRP step was skipped for Cathepsin B as the detection antibody from the kit was already HRP-conjugated. The plate was washed three times with PBST (0.1% Tween 20 in PBS) in-between incubations. In addition, the cathepsin B plate was washed three times with PBST containing 1 M Urea before the incubation with the detection antibody. Following six washes with PBST to remove excess detection antibodies, 100 mL of tetramethyl benzidine (TMB) substrate (0.1 mg/mL, 0.05 M phosphate-citrate buffer, pH 5.0) was incubated in each well at 22 °C. The reaction was stopped within 15–30 min with 50 μL of 2 M H_2_SO_4_, and the data was collected at 450 nm (absorbance filter) using the SpectraFluor reader (Tecan).

### 3.3. Statistical Methods

The data were analyzed using GraphPad Prism (version 4.0C; GraphPad Software, La Jolla, CA). Unpaired *t* test was performed to determine if there was statistical significance between groups. Paired *t*-test was only used to compare the initial and follow-up LDH values from the same patient. All reported *p* values are two-sided. *p* values less than 0.05 were considered statistically significant.

## 4. Conclusions

In summary, our study indicates that serum biomarker studies must be carefully designed to exclude the false influence from non-specific serum antibodies. We have shown that, after the elimination of serum interference, IL-8 and cathepsin B were significantly elevated in melanoma. Additional cytokines/chemokines were also identified as potential serum biomarkers to characterize melanoma. Our studies have provided a basis for the investigation of the change of serum biomarkers (e.g., IL-8 and cathepsin B) as an approach to monitor patients for treatments.

## Figures and Tables

**Table 1.** Comparison of serum IL-8 levels (pg/mL) by groups.

		**N**	**MEAN**	**SD**	**SE**
**Melanoma**					
**Age**					
	**<50**	19	209.1	544.2	124.8
	**50–59**	16	40.5	56.9	14.2
	**60–69**	17	136.6	255.2	61.9
	**≥70**	15	161.5	305.3	78.8
**Stage**					
	**II**	4	20.8	17.6	8.8
	**III**	18	76.8	191.5	45.1
	**IV**	45	175.5	304.7	60.2
**Overall**		67	139.8	347.7	42.5
**Breast Cancer**					
**Overall**		24	76.7	210.3	43.9
**Control**					
**Overall**		13	8.6	6.6	1.8

**Table 2.** Comparison of serum Cathepsin B levels (ng/mL) by groups.

		**N**	**MEAN**	**SD**	**SE**
**Melanoma**					
**Age**					
	**<50**	19	22.7	8.6	2.0
	**50–59**	16	23.4	15.7	3.9
	**60–69**	17	34.5	38.8	9.4
	**≥70**	15	24.4	14.6	3.8
**Stage**					
	**II**	4	18.1	2.9	1.5
	**III**	18	23.6	11.3	2.7
	**IV**	45	28.0	26.5	4.0
**Overall**		67	26.2	22.6	2.8
**Breast Cancer**					
**Overall**		24	38.6	43.5	8.9
**Control**					
**Overall**		13	15.8	8.1	2.2

**Table 3.** Cytokine and chemokine expression in melanoma patients and healthy controls.

**Cytokines**	**Melanoma**	**Control**	*p*
**gm-CSF**	21.9 ± 4.55	8.86 ± 3.2	<0.05
**INF-α**	297 ± 30.7	150 ± 16.9	<0.01
**IL1-α**	4.78 ± 0.93	0.64 ± 0.0	<0.01
**IL1-β**	4.87 ± 1.75	0.064 ± 0.0	<0.05
**IL-7**	395 ± 119	6.72 ± 3.52	<0.01
**IL-8**	1083 ± 417	7.8 ± 2.34	<0.05
**IP-10**	985.3 ± 98.5	741.1 ± 14.29	<0.05
**MIP-1β**	199.7 ± 51.95	46.88 ± 15.60	<0.05
**TNFα**	25.9 ± 5.03	11.6 ± 3.14	<0.05
